# Experimental infection of a newly emerging Korean type I porcine reproductive and respiratory syndrome virus isolate in colostrum-deprived pigs

**DOI:** 10.1186/1743-422X-8-177

**Published:** 2011-04-17

**Authors:** Hye Kwon Kim, Chul Seung Lee, Bo Kyu Kang, Min Ju Yeom, Hyoung Joon Moon, Seong Jun Park, Van Giap Nguyen, Dae Sub Song, Bong Kyun Park

**Affiliations:** 1 Department of Veterinary Medicine Virology Lab, College of Veterinary Medicine and BK21 Program for Veterinary Science, Seoul National University, Gwanak-gu, Seoul, 151-742, Korea; 2 Research Unit, Green Cross Veterinary Products, 227-5, Kugal-dong, Kiheung-gu, Yongin, 449-903, Republic of Korea; 3 Chungang University School of Medicine, Seoul 156-756, Republic of Korea; 4 Viral Infectious Disease Research Center, Korea Research Institute of Bioscience and Biotechnology, Daejon, 305-806, Korea

**Keywords:** type I PRRSV, Korea, Experimental, infection, emerging

## Abstract

**Background:**

Recently, new emergence of type I PRRSV has been reported in Korea by several research groups. Although specific subgroups of type I PRRSVs in Korea were observed in the previous phylogenetic analysis, there is a lack of information about the virulence of type I PRRSV recently isolated in Korea.

**Methods:**

One type I PRRSV isolate (G2446, 3 times passaged in primarily cultured pulmonary macrophages) in Korea was experimentally infected in colostrum-deprived pigs. The pathological and serological evaluations were performed and compared to type II PRRSV strain (CP07-401-9, 5 times passaged in MARC-145 cell lines)-infected pigs, for 21 days post challenge (dpc).

**Results:**

The pneumonia found in gross examination was more severe in type I PRRSV-infected pigs than type II PRRSV-infected pigs. Both groups showed bronchointerstitial pneumonia, mild multifocal perivascular lymphohistiocytic myocarditis and lymphadenopathy at 14 dpc. However, the unique histopathologic lesions were not found in the pigs experimentally infected with a Korean type I PRRSV isolate, when compared to previous data about classical pathology of PRRSV. The PRRS-specific antibodies were detected in the first week after challenge and viremia continued at least until 21 dpc in both groups.

**Conclusion:**

The gross and histopathologic lesion in this study indicated that Korean type I PRRSV strain (G2446) caused classical PRRSV-specific lesions. Although this study evaluated one representative strain of Korean type I PRRSV, the results may provide information regarding the pathogenicity of type I PRRSV recently emerged in Korea.

## Background

Porcine reproductive and respiratory syndrome (PRRS) has spread worldwide and continues to be one of the most devastating diseases of swine throughout the world. PRRS is caused by a small, enveloped, positive strand RNA virus, PRRS virus (PRRSV), which belongs to the family Arteriviridae, genus Arterivirus [1]. Genetic and antigenic analyses have revealed two distinct PRRSV groups, the European (Type I) and the North American (Type II), with marked genetic and antigenic differences between the two genotypes as well as among viruses within each genotype [2-5].

PRRS has been experimentally induced with cell-culture-propagated virus in sows and pigs [6-8]. Also, it has been documented that PRRSV strains differ in virulence [9].

In the Republic of Korea, type II PRRSV infection was first described in 1993 [10]. Since then, there have been studies on the molecular characterization of type II PRRSV [11,12]. Recently, type I PRRSV infection occurred in Korean swine farms, and they showed unique characteristics in genetic analysis [13-16]. The type II PRRSV in Korea was suspected to be introduced from North America, and at least 4 different lineages of type II PRRSV were circulating in Korea [13]. In the nation-wide study, the Korean type I PRRSV (a term used to indicate type I PRRSVs in Korea) formed three distinct clusters from other type I PRRSV strains and cluster I was a predominant group [13,14]. Although the type I PRRSVs in Korea were included in panEuropean subgroup, they were further divided into three clusters (class I, II and III) in the phylogenetic analysis [4,14,15]. The class I was shown to be dominant strains in Korea. However, in spite of nation-wide phylogenetic analysis of the viruses, there is a lack of information about the virulence of type I PRRSV recently isolated in Korea. The aim of this study was to observe gross lesion, histopathological lesion and immunological properties in pigs after experimental infection of a type I PRRSV isolate especially belonged to 'class I', a dominant type I PRRSV in Korea.

## Methods

### Cells and viruses

In the case of type I PRRSV, tissue-culture-infective doses (TCID) were prepared as follows. A G2446 strain (Passage 3 in Pulmonary alveolar macrophages (PAM)) was prepared to viral concentration of 10^5 ^TCID_50_/ml using Dulbecco's modified Eagle's medium (DMEM) with 5% fetal bovine serum (FBS), penicillin (100 units/ml), streptomycin (100 μg/ml) and amphotericin B (0.25 μg/ml). In the case of type II PRRSV, a CP07-401-9 strain (Passage 5 in MARC-145 cells) was prepared to 10^5 ^TCID_50_/ml in the same media as described above for the Type I strain. The viruses were isolated from pigs in Korea and their sequence information was presented in the previous papers [13,15].

### Experimental design

Two ml of 5 logTCID_50_/ml type I and type II PRRSV isolates, G2446 (GenBank ID: GU325647, p3, cluster I) and CP07-401-9 (GenBank ID: FJ972728, p5, vaccine-like), were inoculated into five colostrum-deprived pigs (3 weeks old) for each viral type via the intranasal route. Three pigs remained uninfected as a control group. Each group was maintained in a separate pen. After challenge, blood samples were collected at 4, 5, 6, 7, 8, 9, 10, 11, 12, 14 and 21 days post challenge (dpc). All animal experiments complied with the current laws of South Korea. Animal care and treatment were conducted in accordance with guidelines establishment by the Green Cross Veterinary Products Institutional Animal Care and Use Committee.

### Serological tests

A commercial kit (HerdChek PRRS 2XR, IDEXX Inc., USA) was used, according to the manufacturer's instructions, to estimate the levels of PRRSV-specific antibodies. The IgG titer for PRRSV was indicated as S/P ratio which was the calculated value in the formula: (Sample O.D - Negative control O.D)/(Positive control O.D - Negative control O.D).

A commercial real time polymerase chain reaction kit (Ambion, USA) was used to detect viremia from sera during the study. To evaluate the serum neutralizing ability of both types of viruses, a Serum neutralizing (SN) test was performed based on the previous study [17]. In the case of a type I PRRSV isolate (G2446), PAM cells were used instead of MARC-145 cell lines.

### Gross and histopathological tests

Pigs were euthanized to observe gross and histopathologic lesions at 14 and 21 dpc, respectively, as follows; three and two pigs from the challenged groups, and two and one pigs from the control group. The lung, heart, liver, spleen, kidney, ileum, and lymph nodes were collected and fixed with 4% formaldehyde solution. The RT-nested PCR was performed from the collected organs following previous paper [18]. The fixed organs were processed for histopathology, using hematoxylin and eosin staining.

## Results and discussion

**Figure 1:**
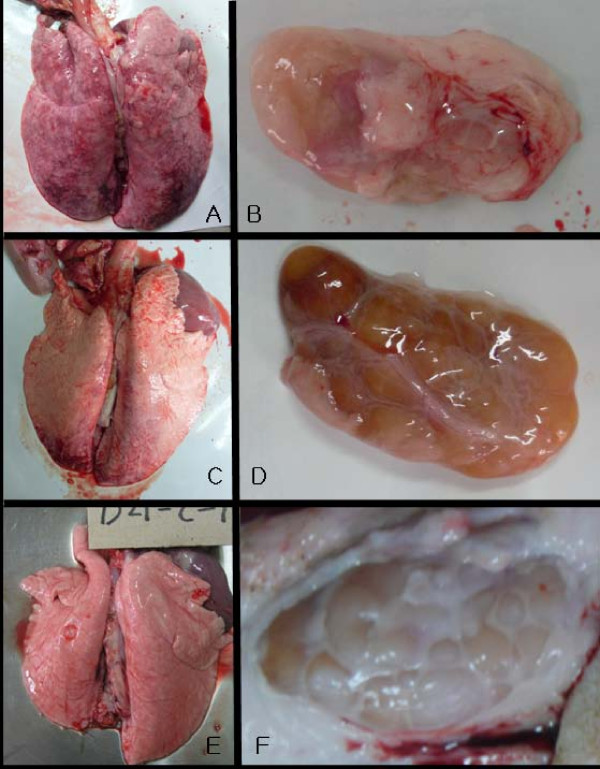
**The gross lesions of lungs and superficial inguinal lymph nodes in the challenged pigs at 14 dpc**. A, lung of G2446 (type I)-challenged group; B, superficial inguinal lymph node of G2446(type I)-challenged group; C, lung of CP07-401-9 (type II)-challenged group; D, superficial inguinal lymph node of CP07-401-9 (type II)-challenged group; E, lung of control group at 21 dpc; F, superficial inguinal lymph node of control group at 21 dpc

Gross lesions were enlarged tan lymph nodes and abnormal lungs in both challenged groups. In the lungs, the parenchyma was mottled or diffusely tan-red in the type I PRRSV-challenged pigs, while only mild regional mottling was observed in the type II PRRSV-challenged pigs (Figure 1).

Histopathological lesions were only found in lung, lymph node and heart tissue (Table 1). The lungs of both challenged groups shared the same pathological observations as follows;

**Table 1:** Histopathological findings in the challenged pigs

DPC*	Type	Pathological findings†		
		
		Lung	Inguinal Lymph Nodes	Heart
14	I	Moderate focal/multifocal bronchointerstitial pneumonia (3/3)	Lymphadenopathy (3/3)	Mild multifocal perivascular lymphohistiocytic myocarditis (1/3)
	
	II	Moderate diffuse/multifocal bronchointerstitial pneumonia (2/3)	Lymphadenopathy (3/3)	Mild multifocal perivascular lymphohistiocytic myocarditis. (3/3)
	
	Control	No lesions (2/2)	No lesions (2/2)	No lesions (2/2)

21	I	Moderate diffuse interstitial pneumonia (2/2), moderate peribronchiolar and perivascular lymphohistiocytic cuffing (2/2)	Lymphadenopathy (2/2)	Mild multifocal perivascular lymphohistiocytic myocarditis (1/2)
	
	II	Mild to moderate diffuse interstitial pneumonia (2/2), severe peribronchiolar and perivascular lymphohistiocytic cuffing (1/2)	Lymphadenopathy (2/2)	Very mild multifocal perivascular lymphohistiocytic myocarditis (1/2)
	
	Control	No lesions (1/1)	No lesions (1/1)	No lesions (1/1)

* days post challenge

† No lesions were observed in liver, spleen, kidney and ileum.

(1) bronchointerstitial pneumonia, which was characterized by lymphohistiocytic inflammation and an accumulation of necrotic cells, likely originated from alveolar macrophages, in alveolar spaces, and (2) Type 2 pneumocyte hyperplasia in the alveolar septa. Mild multifocal perivascular lymphohistiocytic myocarditis was sometimes observed in both challenged groups as well. In the control group, there were no pathologic changes. The results of RT-nested PCR showed PRRSV-positive in all collected organs in both challenged groups during the study (data not shown).

**Figure 2:**
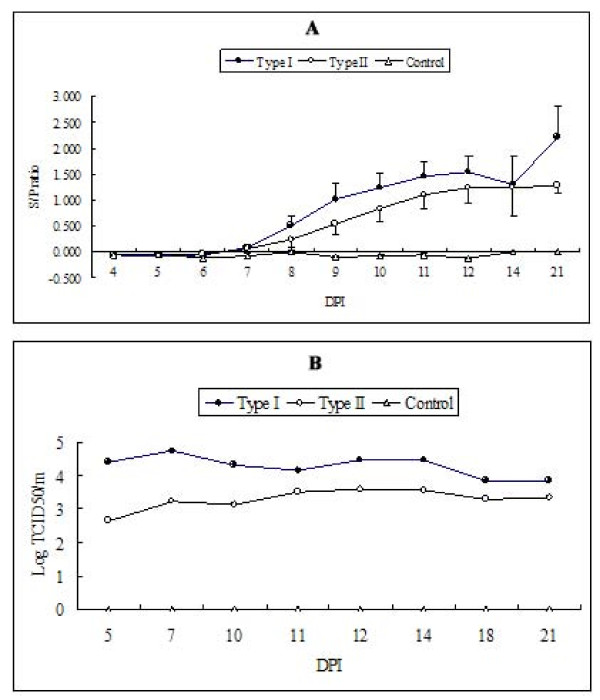
**PRRSV-specific serum IgG (A) and viremic titers (B)**. Error bars in type I and II-challenged groups indicate standard errors. For the quantitation of viremia, a commercial real time RT-PCR kit was used and virus titer was calculated using a standard curve generated from serially diluted type I or II virus isolates. Two serum samples from each dpc collection were stored until 21 dpc. At this point, they were pooled and quantitative RT-PCR was performed. Since no viral genome was detected in the control group, viral titer in control group sera was indicated as '0'.

PRRSV-specific antibodies were first detected at 8 and 9 dpc in type I and type II PRRSV-challenged groups, respectively (Figure 2). Although it was not significant by Student's T test, mean s/p ratios remained higher in the type I PRRSV-challenged group than in the type II challenged group during the study. Even in the presence of PRRSV-specific IgG, no detectable level of SN titer was observed in either challenge group, and viremia was also maintained throughout the study.

In this study, the virulence of newly emerging type I PRRSV in Korea was evaluated in young pigs, using one type I PRRSV isolate. Our main aim was to evaluate the virulence of newly emergent type I PRRSV under experimental conditions. Although only one isolate of type I PRRSV was used in this study, the data may provide fundamental information for further comparative studies. Since the previous study had reported the observation of PRRSV-specific histopathological lesions from 3 to 21 dpc, we tested the challenged pigs until 21 dpc [19]. Previous pathogenicity study of type II PRRSV isolated in Korea showed that alveolar proteinaceous and karyorrhectic debris were interspersed with macrophages as well as mild type-II pneumocyte hyperplasia and hypertrophy [19]. The comparative study using two type II PRRSV and one type I PRRSV also revealed that both types of PRRSVs could induce lymphohistiocytic myocarditis, lymphadenopathy and encephalitis as well as lung lesions [9].

In agreement with the previous data, pathologic changes after experimental challenge with a Korean isolate of type I PRRSV (class I) were observed in the lungs, lymph nodes and heart, until 14 and 21 dpc. The pathologic changes by the Korean isolate of type I PRRSV in this study were also type 2 pneumocyte hyperplasia, necrotic debris in alveolar septa, perivascular lymphohistiocytic myocarditis and lymphadenopathy, which were all similar to previous reports. These data indicated that a type I PRRSV isolate, in this study, showed classical lesions of PRRSV infection and sufficiently induced acute disease in young pigs (3 weeks old). Pigs at this age are known to be in a vulnerable stage for PRRSV infection [20]. Although unique lesions of the Korean isolate of type I PRRSV was not found in this study, the severity of gross lesions in lungs was higher than that of the type II PRRSV strain (VR-2332-like). Therefore, type I PRRSV (G2446, class I) in this study may also present a risk for co-infections with other viruses and bacteria in the pigs during the nursery to growing period.

The seroconversion was also well-defined by ELISA around 8 dpc. Although mean S/P ratio was higher in type I PRRSV challenged pigs, it was not significant by student's T test, which led incapable of differentiating type I and II PRRSV infection in the field using IDEXX ELISA. Further consideration will be needed for type-specific serological methods to differentiate type I and II PRRSV infection in Korea. In this study, no SN titer was observed until 21 dpc in both group. Since SN titer for type I PRRSV was usually detected at around 35 days post inoculation, the lack of detection of SN antibodies in this study is probably due to the short period of time of experiment (21 days) [21]. Furthermore, the prolonged viremia of both types I and II PRRSV observed in this study raised concerns about their long-term transmission among pigs. Therefore, type-specific PRRSV surveillance and control policy are important to minimize the type I PRRSV transmission in Korea.

In conclusion, the Korean isolate of type I PRRSV used in this study could induce classical PRRSV-specific lesions and serological properties. Although only one strain of Korean type I PRRSV was evaluated in this study, the results could provide information about the virulence of recently emerging Korean type I PRRSV. However, the virulence of PRRSV infection could be differed among the same types of PRRSV strains [9]. So, further comparative studies based on this study are needed to be followed.

## Competing interests

The authors declare that they have no competing interests.

## Authors' contributions

HKK: Attending the experiments and writing a manuscript, CSL: Attending the experiments and sample collection, BKK: Organized the animal experiment in the facility, MJY: conducted animal care and sample collection, HJM: conducted animal care and sample collection, SJP: Attributed to statistical analysis, VGN: participated writing a manuscript for introduction, DSS: Idea development and designing all experiments, BKP: final correction of the manuscript. All authors read and approved the final manuscript.
